# Draft Genome Sequence of Zhouia amylolytica CL16, Isolated from Mangrove Soil

**DOI:** 10.1128/mra.00068-23

**Published:** 2023-04-05

**Authors:** Clarine Wan Ling Hong, Kok Jun Liew, Ming Quan Lam, Chun Shiong Chong

**Affiliations:** a Department of Biosciences, Faculty of Science, Universiti Teknologi Malaysia, Skudai, Johor, Malaysia; b Department of Biological Science, Faculty of Science, Universiti Tunku Abdul Rahman, Kampar, Perak, Malaysia; Loyola University Chicago

## Abstract

Zhouia amylolytica CL16 was isolated from the mangrove soil of Tanjung Piai, Malaysia. The present work reports the draft genome sequence of this bacterium. The genome consists of 113 glycoside hydrolases, 40 glycosyltransferases, 4 polysaccharide lyases, 23 carbohydrate esterases, 5 auxiliary activities, and 27 carbohydrate-binding modules, which warrant further investigation.

## ANNOUNCEMENT

*Zhouia* spp. are halophilic bacteria that live in saline habitats (1–3). At the time of writing, Zhouia amylolytica and Zhouia spongiae have been documented in the List of Prokaryotic Names with Standing in Nomenclature (LSPN). These two species were isolated from marine samples (e.g., sediments and sponge) (1–3). The origin of Zhouia amylolytica CL16 was mangrove soil, which differs from the aforementioned species. Here, the draft genome sequence of strain CL16 was reported to reveal the genomic information and compare it with the reported *Zhouia* spp.

Zhouia amylolytica CL16 was isolated from mangrove soil at Tanjung Piai, Johor, Malaysia (1°16′06.0″N, 103°30′31.2″E). One gram of soil sample was resuspended in 10 mL sterile distilled water and then serially diluted and plated on marine agar (BD Difco, USA). After 24 h of incubation at 37°C, colonies from the spread plates were subcultured to achieve a pure culture. The colony of strain CL16 was grown in marine broth (BD Difco, USA) for 18 h at 37°C and 150 rpm. Its genomic DNA was extracted using Quick-DNA miniprep Plus kit (Zymo Research, USA) following the manufacturer’s protocol. Then, PCR with universal primers (27F and 1492R) was used to amplify the 16S rRNA gene sequence of the bacterium (4). The amplified product was sequenced via Sanger sequencing, and the result was BLASTN searched against NCBI standard database (nucleotide collection). The extracted genomic DNA was then used for library preparation via the Nextera XT library preparation kit. Whole-genome sequencing was accomplished by paired-end sequencing (2 × 250 bp) using the Illumina MiSeq platform. After the sequencing, the adapter and low-quality sequences were filtered with BBTools version 36 (parameters, trimq=30 ref=phix.fa minlength=50) (5). *De novo* assembly was completed through SPAdes version 3.9.0 (parameters, --careful -k 21,33,55,67) (6). The genome was then annotated using NCBI Prokaryotic Genome Annotation Pipeline (PGAP) version 5.0 (7). The dbCAN2 meta server was used for carbohydrate-active enzymes (CAZymes) prediction (8). The CAZymes were verified via BLASTP against nonredundant protein sequences (nr) and Swiss-Prot databases (9, 10). The average nucleotide identity (ANI) and digital DNA-DNA hybridization (dDDH) were determined via EzBioCloud ANI calculator and Type (Strain) Genome server (11, 12). Unless specified, default parameters were used.

The 16S rRNA gene sequence of strain CL16 showed 95.83% to 100% sequence identity with other *Zhouia* spp., indicating that it belongs to the *Zhouia* genus. The Illumina sequencing generated 2,611,216 raw paired-end reads. Upon removing the low-quality reads, the clean reads were assembled into 84 contigs, consisting of a total length of 3,781,374 bp with 184.08× coverage, GC content of 36.8%, and an *N*_50_ value of 167,753 bp. A total of 3,276 genes are encoded by strain CL16. These include 3,219 protein-coding genes, 3 rRNA genes (5S, 16S, and 23S rRNA), 41 tRNA genes, 4 noncoding RNA genes, and 9 pseudogenes. The ANI values of strain CL16 against Z. amylolytica strains CGMCC 1.6114 (assembly accession no. GCA_900116365) and AD3 (assembly accession no. GCA_000511935) and Z. spongiae strain HN-Y44 (assembly accession no. GCA_022760175) were found to be 99.28%, 98.72%, and 76.77%, respectively, whereas the dDDH values were found to be 93.70%, 88.70%, and 19.90%, respectively. Collectively, strain CL16 is likely the same species as Zhouia amylolytica.

The mangrove environment consists of lignocellulosic plant biomass. The bacteria residing in this environment may possess lignocellulolytic genes for their survival. Based on CAZymes analysis, strain CL16 encodes 113 glycoside hydrolases (GHs), 40 glycosyltransferases (GTs), 4 polysaccharide lyases (PLs), 23 carbohydrate esterases (CEs), 5 auxiliary activities (AAs), and 27 carbohydrate-binding modules (CBMs). Among the CAZymes, some were annotated as acetylxylan esterase, α-glucosidase, α-l-fucosidase, β-galactosidase, α-1,2-mannosidase, β-glucosidase, β-xylosidase, cellulase, and xylanase. This result shows that strain CL16 has the potential to degrade lignocellulose material.

### Data availability.

The 16S rRNA gene sequence of Zhouia amylolytica CL16 has been deposited in NCBI GenBank with accession number OP363861. The draft genome sequence of Zhouia amylolytica CL16 has been deposited in DDBJ/ENA/GenBank under accession number JAEMBF000000000, BioProject accession number PRJNA687284, and BioSample accession number SAMN17140992. The raw sequence read files of Zhouia amylolytica CL16 have been deposited in the NCBI Sequence Read Archive under accession number SRR22764708.
